# Editorial: Novel Risk Predicting System for Heart Failure

**DOI:** 10.3389/fcvm.2022.954341

**Published:** 2022-06-13

**Authors:** Chen Liu, Nicholas Cauwenberghs, Ning Zhou

**Affiliations:** ^1^Department of Cardiology, The First Affiliated Hospital of Sun Yat-sen University, Guangzhou, China; ^2^Research Unit of Hypertension and Cardiovascular Epidemiology, Department of Cardiovascular Sciences, Katholieke Universiteit (KU) Leuven, Leuven, Belgium; ^3^Tongji Hospital, Tongji Medical College, Huazhong University of Science and Technology, Wuhan, China

**Keywords:** risk prediction, imaging, genetics, heart failure, circulating biomarkers

The prevalence of heart failure (HF) is a major public health problem, as its prevalence and high morbidity and mortality are increasing worldwide. To date, we are in need of biomarkers that improve the diagnosis and prognosis of HF. Within this context, this Research Topic called for research on novel HF risk factors as diagnostic and prognostic cues that may improve HF detection and prognosis. Ideally, a HF marker should reflect pathological perturbations that contribute to HF pathogenesis and progression, while enabling prediction, diagnosis, staging, prognosis or management of this condition. As represented in this Research Topic, HF biomarkers may include easily accessible clinical parameters as well as more advanced biomarkers involving (epi)genetics, imaging and blood biochemistry.

For instance, Li Y. et al. investigated the association between fluctuations in a basic clinical parameter (body weight) and the risk of adverse events in 1,691 patients with HF with preserved ejection fraction (HFpEF). In this TOPCAT cohort, higher body weight variability over time was associated with higher risk of future cardiovascular events independent of traditional risk factors and regardless of the direction of the weight change. As such, body weight monitoring may improve risk stratification in patients with HFpEF.

In search for genetic markers of HF, Li X. et al. conducted a Mendelian randomization study on inflammatory biomarkers in HF. They collected genome-wide association study data of 47,309 HF cases and 930,014 controls of European descent to identify genetic variants in inflammatory biomarkers such as C-reactive protein (CRP) and fibrinogen that may underlie HF. Overall, none of the SNPs for CRP, fibrinogen and components of the interleukin-1 and −6 signaling pathway were causally linked to HF risk. More studies are warranted to identify (epi)genetic markers associated with HF pathogenesis and progression.

Furthermore, this Research Topic includes three original research articles on the predictive value of imaging markers in a hospital setting. First, Yang et al. followed 156 patients with concomitant HF with reduced ejection fraction (HFrEF) and atrial fibrillation who underwent first-time catheter ablation. One year after ablation, left ventricular ejection fraction (LVEF) was improved in 72.3% of patients, which could only be predicted by E/e', an echocardiographic surrogate marker of LV filling pressure [OR_adjusted_ 1.13 (1.03–1.24); *P*_adjusted_ = 0.011]. E/e'≥15 provided optimal prediction with poor sensitivity (38.7%) but high specificity (89.2%). E/e' may thus complement current risk prediction approaches in HFrEF patients and atrial fibrillation undergoing catheter ablation.

Furthermore, Zhu et al. conducted a case-control study investigating non-invasive markers of myocardial work (MW, calculated from pressure-strain loops using 2D speckle tracking echocardiography). In adjusted analyses, MW indexes strongly correlated with LVEF and differed between patients with coronary artery disease (CAD) and healthy controls. Future studies should dig deeper into the additive value of MW indexes beyond conventional echocardiographic parameters of LV structure and systolic and diastolic function for risk stratification and diagnosis in CAD and HF.

Zhang et al. monitored heart-rate recovery at 1 min after exercise (HRR1) in 89 consecutive patients with inoperable chronic thromboembolic pulmonary hypertension undergoing balloon pulmonary angioplasty (BPA). In this study, HRR1 significantly improved after the procedure, suggesting the alleviation of sympathovagal imbalance upon BPA. HRR1 may thus represent an easily available and non-invasive surrogate marker to predict BPA outcome and monitor its efficacy.

Besides genetic and imaging markers, also circulating biomarkers may enable early detection of HF and pave the way to novel therapies. Two state-of-the-art reviews in this Research Topic illustrate the potential of circulating biomarkers in HF. One review on cardio-oncology provides a thorough overview of the advancements in the field of biomarkers to monitor cardiovascular toxicity of various tumor therapies. The review particularly focuses on subclinical markers of cancer treatment-related cardiac dysfunction during drug therapy and radiotherapy. Another review summarizes the current literature on the relationship between the amino-acid metabolism and gut microbiome alterations during the development of heart failure, while describing the potential prognostic and therapeutic value of the gut–amino acid–HF axis. In line, four original research studies in this Research Topic elaborated on the value of: (i) α-linolenic acid for HF risk stratification (a meta-analysis), (ii) plasma galectin-3 for diagnostic and prognosis of HFrEF, (iii) insulin growth factor-1 (IGF-1) and IGF-binding protein 1 for detection of HF subtypes, and (iv) the systemic immune-inflammation index for prediction of short-term mortality in overt HF. In addition, Xu et al. dug deeper in the molecular mechanisms underlying the development of cardiac hypertrophy. In particular, they found that Samm50 can promote cardiac hypertrophy by regulating Pink1-Parkin-mediated mitophagy. This pathway may be an interesting therapeutic target for managing cardiac hypertrophy. Each of these studies highlight the potential for circulating biomarkers to complement current strategies used in clinic for stratifying a patient's risk for HF and assessing HF prognosis. The findings on circulating biomarkers may even pave the way to novel therapeutic options for HF as illustrated by the study on Samm50. Evidently, future studies should validate the findings, investigate which and how these novel biomarkers could be implemented cost-effectively in HF clinic and investigate therapeutic strategies linked to the highlighted pathways contributing to HF pathology.

For clinical practice, multidimensional risk scores will be inevitable, as no single HF biomarker will fulfill all clinical needs (diagnosis, prediction, prognosis, monitoring, etc.) on its own. Within this context, Gong et al. tested the risk stratification strategy for pulmonary arterial hypertension as endorsed by European guidelines in 392 patients with idiopathic pulmonary arterial hypertension in China. Of note, the multidimensional risk stratification approach effectively stratified along risk, while accurately predicting mortality in these patients. Therapeutic implications of this risk grading approach remain to be resolved. Furthermore, Yan et al. trained and tested a nomogram score from eight preoperative factors to predict the risk of in-hospital mortality in HFrEF patients after coronary artery bypass grafting surgery (CABG). While the EuroSCORE-2 underestimated postoperative mortality risk, especially in high-risk patients, their nomogram provided better preoperative prediction of mortality after CABG in patients with HFrEF. This may facilitate identifying HFrEF patients at high risk of post-procedural in-hospital mortality.

In the future, optimal HF management will require integration of the most informative biomarkers, regardless of whether they form clinical, (epi)genetic, imaging or biochemical evidence of HF pathology. Thus, future studies should identify the ideal combination of biomarkers that captures the full spectrum of HF pathogenesis and progression and that adequately steers the clinical decision-making process.

## Author Contributions

All authors have made a substantial contribution to the concept or design of the editorial, either drafted the article or critically revised it critically for important intellectual content, approved the submitted version to be published, and agreed to be accountable for all aspects of the work.

## Conflict of Interest

The authors declare that the research was conducted in the absence of any commercial or financial relationships that could be construed as a potential conflict of interest.

## Publisher's Note

All claims expressed in this article are solely those of the authors and do not necessarily represent those of their affiliated organizations, or those of the publisher, the editors and the reviewers. Any product that may be evaluated in this article, or claim that may be made by its manufacturer, is not guaranteed or endorsed by the publisher.

